# Predicting length of hospital stay in community-acquired pneumonia using clinical and treatment factors: a retrospective study with restricted cubic spline and piecewise regression analysis

**DOI:** 10.3389/fpubh.2026.1768432

**Published:** 2026-04-22

**Authors:** Hualong Zeng, Chengqing Yang, Min Jiang, Xiaohui Luo, Genxiu Luo, Xianyang Chen, Yige Song, Lei Wang, Xiaojun Zhu, Xiaomin Zheng, Hong Wei, Juewei Pan, Feng Lin

**Affiliations:** 1 Sanming First Hospital Affiliated to Fujian Medical University, Sanming, China; 2 Biomedical Center, Zhongguancun Big Data Industry Alliance, Beijing, China; 3 Department of Neurology, Beijing Chaoyang Hospital, Capital Medical University, Beijing, China

**Keywords:** antibiotic duration, community-acquired pneumonia, length of stay, non-linear modeling, restricted cubic spline

## Abstract

**Background:**

Community-acquired pneumonia (CAP) remains a leading cause of hospitalization worldwide. Accurate prediction of length of stay (LOS) is crucial for optimizing hospital bed turnover, improving clinical resource allocation, and facilitating the development of individualized discharge plans, thereby reducing the strain on healthcare systems.

**Methods:**

We retrospectively analyzed 423 adults hospitalized with CAP from January 2022 to December 2023. Clinical characteristics, laboratory data, comorbidities, and treatment variables were extracted from electronic health records. Univariate and multivariable linear regression models were initially employed to identify independent predictors of LOS. The predictive performance of the final multivariable model was assessed using R^2^, adjusted R^2^, Akaike Information Criterion, and 10-fold cross-validation. To further explain the complex relationship between specific treatment factors and LOS, restricted cubic splines and piecewise regression were utilized, specifically to evaluate non-linear associations and identify clinical inflection points.

**Results:**

The final model showed strong performance (R^2^ = 0.864; adjusted R^2^ = 0.862). Independent predictors of prolonged LOS included respiratory failure, pressure ulcers, elevated blood urea nitrogen, antibiotic modification during hospitalization, traditional Chinese medicine use, and antibiotic duration. Restricted cubic spline analysis demonstrated a significant non-linear relationship between antibiotic duration and LOS (*P* < 0.05). Piecewise regression identified an inflection point at 7.398 days, after which LOS increased more rapidly.

**Conclusions:**

Multiple clinical and treatment-related factors were associated with LOS in CAP. Antibiotic duration showed a pronounced non-linear pattern, with treatment beyond 1 week linked to markedly longer hospitalization. These findings may help identify patients at risk of prolonged LOS and support more efficient clinical decision-making.

## 1 Introduction

Community-acquired pneumonia (CAP) is one of the most common causes of acute respiratory infection worldwide and continues to impose a substantial burden in terms of morbidity, hospitalization, and mortality. CAP affects individuals across all age groups, but its impact is particularly pronounced among older adults. Large national and international surveillance studies have consistently shown that the risk of severe disease, complications, and death increases sharply with advancing age (1). Recent analyses also indicate that adults aged ≥65 years' experience disproportionally higher rates of pneumococcal and viral CAP, more frequent atypical or nonspecific presentations, and a greater likelihood of respiratory failure or intensive-care admission (2, 3). Age-related declines in immune function and the high prevalence of chronic comorbidities further contribute to more severe clinical courses and prolonged recovery in this population.

The length of hospital stay (LOS) is an important indicator that reflects disease severity, clinical management efficiency, and healthcare resource utilization. Prior studies have reported average LOS ranging from 5–7 days in non-severe CAP and 7–14 days in severe cases (4), while retrospective cohorts commonly describe a median LOS of approximately 7–11 days (5). Extended hospitalization is associated with increased medical costs and reduced bed turnover, placing a substantial burden on healthcare systems.

Although several LOS prediction models for CAP have been proposed—including those by Suter-Widmer et al. and Uematsu et al.—their clinical applicability remains limited (6, 7). While some existing models demonstrate adequate discrimination within their original derivation cohorts, their transportability to different healthcare settings remains uncertain. This limitation largely stems from a failure to account for regional variations in treatment protocols, including the integration of Traditional Chinese Medicine. Furthermore, these tools rely predominantly on static admission baseline data and suffer from missing dynamic variables. Crucial factors like in-hospital antibiotic adjustments and precise treatment duration are frequently omitted. Finally, relying solely on strict linear assumptions can compromise model calibration. Such linear models erroneously imply a constant, proportional increase in LOS for each additional day of treatment. In clinical reality, the relationship is highly non-linear: while the initial days of antibiotic therapy typically reflect standard, uncomplicated CAP management, a prolonged duration often signifies treatment failure, resistant pathogens, or severe in-hospital complications. These adverse events lead to a disproportionately steeper escalation in hospitalization time, which strict linear models fundamentally fail to capture.

To address these gaps, this study aimed to develop a LOS prediction model for hospitalized CAP patients using routinely available clinical and treatment-related data collected at admission and during hospitalization. By applying restricted cubic splines and piecewise regression, the study further explored the non-linear association between antibiotic duration and LOS. The objective was to provide an interpretable and clinically relevant tool that may help optimize treatment strategies and improve the allocation of healthcare resources for CAP management in China.

## 2 Methods

### 2.1 Research design and participants

This retrospective study included consecutive adult patients diagnosed with community-acquired pneumonia (CAP) who were admitted to the First Hospital of Sanming City between January 1, 2022, and December 31, 2023. Patients were identified using International Classification of Diseases (ICD) codes related to pneumonia. To minimize misclassification bias, this initial electronic screening was followed by a manual review of electronic health records. We clinically validated each CAP diagnosis by confirming the presence of acute respiratory symptoms and corresponding radiographic or laboratory findings documented at admission. Cases lacking clear clinical evidence or suggesting alternative diagnoses were excluded. A total of 423 eligible cases were included.

The study was conducted as part of an institutionally approved research project, for which ethical approval was obtained prospectively in April 2021. The study protocol was reviewed and approved by the Ethics Review Committee of the First Hospital of Sanming City [No. Mingyiyuan Ethics (2021) No. 63]. This approval covered the collection and subsequent retrospective analysis of anonymized clinical data generated during the project period. All procedures complied with relevant guidelines and regulations. The requirement for informed consent was waived owing to the retrospective design.

Inclusion criteria were: (1) age ≥18 years; (2) diagnosis of CAP based on clinical symptoms and radiographic or laboratory evidence; (3) first hospitalization for CAP during the study period; (4) hospitalization primarily for CAP management.

Exclusion criteria were: (1) immunodeficiency; (2) lung malignancy or active pulmonary tuberculosis; (3) hospital-acquired pneumonia; (4) chronic underlying lung diseases (e.g., COPD, bronchiectasis, asthma); (5) concomitant stroke; (6) pre-admission disease course >30 days; (7) in-hospital death.

Patients with chronic underlying lung diseases were specifically excluded to minimize confounding bias. Respiratory infections in these populations typically trigger acute exacerbations that require distinct pharmacological interventions (such as systemic corticosteroids or bronchodilators). These concurrent treatments independently alter length of stay, which would obscure the specific impact of CAP and its corresponding antibiotic duration. The sample size was determined by all eligible consecutive cases during the study period.

### 2.2 Data collection and processing

Data were extracted from the electronic health records (EHR) system. Collected variables included demographic characteristics (age and sex), clinical symptoms and vital signs at admission, comorbidities and in-hospital complications, microbiological findings, and treatment-related information such as antibiotic type, changes in antibiotic regimen, duration of antibiotic use, fever duration, and other dynamic in-hospital data. The primary outcome was the length of hospital stay (LOS), defined as the number of days from admission to discharge. Operational definitions for key variables were established to ensure clinical consistency. Respiratory failure was defined by the formal discharge diagnosis, which was corroborated by arterial blood gas analysis and documented clinical evidence. Antibiotic regimen change was strictly defined as the escalation or switching of antibiotic classes due to clinical worsening, explicitly excluding routine de-escalation (such as intravenous-to-oral transitions of the same antibiotic). Traditional Chinese medicine (TCM) use referred exclusively to the administration of oral TCM decoctions or proprietary oral medicines during hospitalization; no intravenous TCM injections were included in this cohort.

All variables included in the final analysis had complete data; therefore, no imputation for missing values was necessary. Continuous variables were examined for distributional characteristics and potential outliers before statistical modeling.

### 2.3 Statistical analysis

Length of hospital stay was treated as a continuous outcome variable. First, univariate linear regression analyses were conducted to examine associations between each variable and LOS. Variables with *P* < 0.05 in univariate analyses or considered clinically relevant were entered into a multivariable linear regression model using a stepwise selection strategy. Model performance was assessed using the coefficient of determination (R^2^) and the Akaike Information Criterion (AIC). Furthermore, residual diagnostics were visually evaluated to assess linear regression assumptions, including homoscedasticity. Additionally, multicollinearity among the independent predictors was assessed using the Variance Inflation Factor (VIF), with a threshold of VIF < 5 indicating the absence of significant collinearity.

To evaluate model stability, 10-fold cross-validation was performed. The dataset was randomly divided into 10 equal subsets; in each iteration, nine subsets were used for training and one for validation. The mean R^2^, mean absolute error (MAE), and root mean square error (RMSE) across the 10 fold were calculated as overall model performance indicators.

Non-linear analysis was conducted to assess the relationship between antibiotic duration and LOS. Restricted cubic spline (RCS) functions with four knots placed at the 5th, 35th, 65th, and 95th percentiles were used to identify potential non-linear associations. When non-linearity was detected, a piecewise (segmented) regression model was applied to estimate the inflection point of antibiotic duration. Model fit was evaluated using R^2^ and residual standard error (RSE).

A two-sided *P* value < 0.05 was considered statistically significant. All statistical analyses were performed using R software (version 4.2.2).

## 3 Results

### 3.1 Participant characteristics

A total of 423 patients diagnosed with community-acquired pneumonia were included in this study (Table 1). The mean age was 63.0 ± 17.2 years, and 248 (58.6%) were male. The average length of hospital stay was 11.8 ± 5.9 days for males and 10.8 ± 4.4 days for females. Most patients presented with at least one comorbidity. The most frequent comorbidities included liver insufficiency (26.0%), cardiac insufficiency (18.7%), renal insufficiency (13.5%). Importantly, clinical severity at admission was captured through objective indicators; specifically, respiratory failure—which typically necessitates advanced respiratory or oxygen support—was present in 6.9% of patients. Less common conditions included acute cardiovascular events (1.4%), extrapulmonary malignancies (4.7%), and pressure ulcers.

**Table 1:** Baseline characteristics of the study population (*N* = 423).

Characteristic	Value
Demographics	
Age, years, mean ± SD	63.0 ± 17.2
Sex, male, *n* (%)	248 (58.6%)
Sex, female, *n* (%)	175 (41.4%)
Comorbidities, *n* (%)	
Liver insufficiency	110 (26.0%)
Cardiac insufficiency	79 (18.7%)
Renal insufficiency	57 (13.5%)
Respiratory failure	29 (6.9%)
Acute cardiovascular events	6 (1.4%)
Extrapulmonary malignancy	20 (4.7%)
Pressure ulcers	14 (3.3%)
Symptoms at admission, *n* (%)	
Cough	401 (94.8%)
Expectoration	369 (87.2%)
Fever	210 (49.6%)
Chills	102 (24.1%)
Shivering	41 (9.7%)
Decreased appetite	149 (35.2%)
Sleep disturbance	67 (15.8%)
Shortness of breath/wheezing	118 (27.9%)
Chest tightness/chest pain	108 (25.5%)
Cyanosis	9 (2.1%)
Vital signs at admission, mean ±SD	
Temperature, °C	36.9 ± 0.8
Pulse, bpm	87.1 ± 15.7
Respiratory rate, breaths/min	21.1 ± 2.1
Systolic blood pressure, mmHg	129.1 ± 20.3
Diastolic blood pressure, mmHg	76.3 ± 11.8
Lung auscultation, *n* (%)	
No rales	78 (18.4%)
Wet rales	330 (78.0%)
Mixed dry–wet rales	15 (3.5%)
In-hospital clinical course	
Duration of antibiotic use, days, mean ± SD	11.5 ± 4.9
Fever duration, days, median (IQR)	3 (1–6)
Pre-admission fever duration, days, median (IQR)	0 (0–3)
Duration of dyspnea/wheezing, days, median (IQR)	2 (0–5)

Common clinical symptoms at admission were cough (94.8%), expectoration (87.2%), fever (49.6%), chills (24.1%), shivering (9.7%), decreased appetite (35.2%), sleep disturbance (15.8%), shortness of breath (27.9%), chest tightness or chest pain (25.5%), and cyanosis (2.1%).

During hospitalization, the mean duration of antibiotic use was 11.5 ± 4.9 days. Additional in-hospital variables included fever duration (median 3 days, IQR 1–6), pre-admission fever duration (median 0 days, IQR 0–3), and duration of dyspnea or wheezing (median 2 days, IQR 0–5). Furthermore, initial disease severity was reflected in vital signs at admission, which included temperature (36.9 ± 0.8 °C), pulse rate (87.1 ± 15.7 bpm), respiratory rate (21.1 ± 2.1 breaths/min), systolic blood pressure (129.1 ± 20.3 mmHg), and diastolic blood pressure (76.3 ± 11.8 mmHg). Lung auscultation revealed no rales in 18.4%, wet rales in 78.0%, and mixed dry–wet rales in 3.5% of patients.

### 3.2 Univariate analysis

Univariate linear regression identified several variables significantly associated with the length of hospital stay. These included the presence of respiratory failure, presence of pressure ulcers, elevated blood urea nitrogen level, change in antibiotic regimen during treatment, prolonged duration of antibiotic use, and the use of traditional Chinese medicine. These variables with statistical significance were selected as candidate predictors for the multivariable analysis.

### 3.3 Multivariable regression model

Significant predictors from the univariate analyses were incorporated into a multivariable linear regression model. Rather than relying solely on *P*-values, the specific effect sizes of independent predictors were explicitly quantified using unstandardized regression coefficients (β) and 95% CI (Table 2). Prolonged duration of antibiotic use (β = 0.97, 95% CI: 0.93 to 1.01), presence of pressure ulcers (β = 1.39, 95% CI: 0.27 to 2.51), traditional Chinese medicine use (β = 1.04, 95% CI: 0.22 to 1.85), change in antibiotic regimen (β = 0.77, 95% CI: 0.10 to 1.43), and elevated blood urea nitrogen (β = 0.14, 95% CI: 0.10 to 0.18) were all independently associated with an extended length of hospital stay. Respiratory failure also remained a significant predictor in this adjusted model (β = −0.85, 95% CI: −1.65 to −0.05). Multicollinearity diagnostics confirmed that all VIFs for the predictors were well below the threshold of 5 (ranging from 1.019 to 1.157), indicating no significant multicollinearity in the final model. The final model demonstrated excellent explanatory performance, with an R^2^ of 0.864 and an adjusted R^2^ of 0.862, indicating strong predictive ability (Table 3). Model parsimony was supported by an Akaike Information Criterion (AIC) of 1794.722. The 10-fold cross-validation procedure showed stable model performance, with consistent R^2^, MAE, and RMSE across validation folds.

**Table 2:** Multivariable linear regression model coefficients for predicting length of hospital stay.

Variables	Coefficient	95% CI	*P*-value
Intercept	−0.70	(–1.22, –0.18)	0.009
Respiratory failure	–0.85	(–1.65, –0.05)	0.037
Pressure ulcers	1.39	(0.27, 2.51)	0.015
Blood urea nitrogen	0.14	(0.10, 0.18)	< 0.001
Antibiotic change	0.77	(0.10, 1.43)	0.023
Duration of antibiotic use	0.97	(0.93, 1.01)	< 0.001
Traditional Chinese medicine use	1.04	(0.22, 1.85)	0.013

CI, confidence interval. A two-sided *P* < 0.05 was considered statistically significant.

**Table 3:** Final multivariable model performance statistics.

Statistic	Value
Number of observations	423
R^2^	0.864
Adjusted R^2^	0.862
Akaike information criterion (AIC)	1,794.722

*R*^2^, coefficient of determination; AIC, Akaike Information Criterion.

### 3.4 Non-linear association between antibiotic duration and length of hospital stay

**Figure 1:**
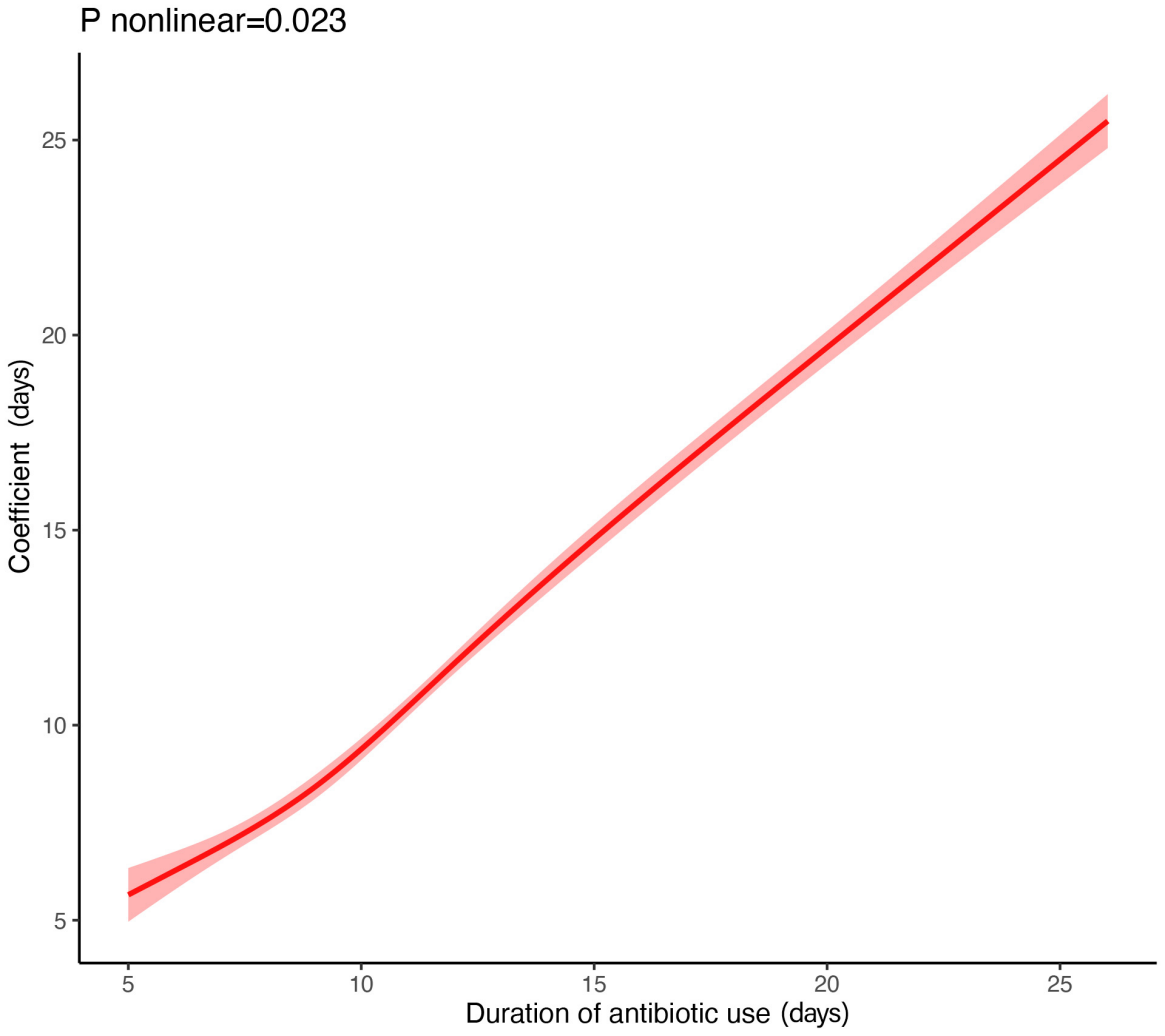
Restricted cubic spline analysis of antibiotic duration and length of stay. Restricted cubic spline curve (four knots) showing a significant non-linear association between antibiotic duration and hospital stay (*P* for non-linearity < 0.05). Shaded band indicates the 95% confidence interval.

Restricted cubic spline (RCS) analysis demonstrated a significant non-linear association between the duration of antibiotic use and length of hospital stay (P for non-linearity < 0.05), revealing a two-segment increasing trend across the exposure range (Figure 1).

**Figure 2:**
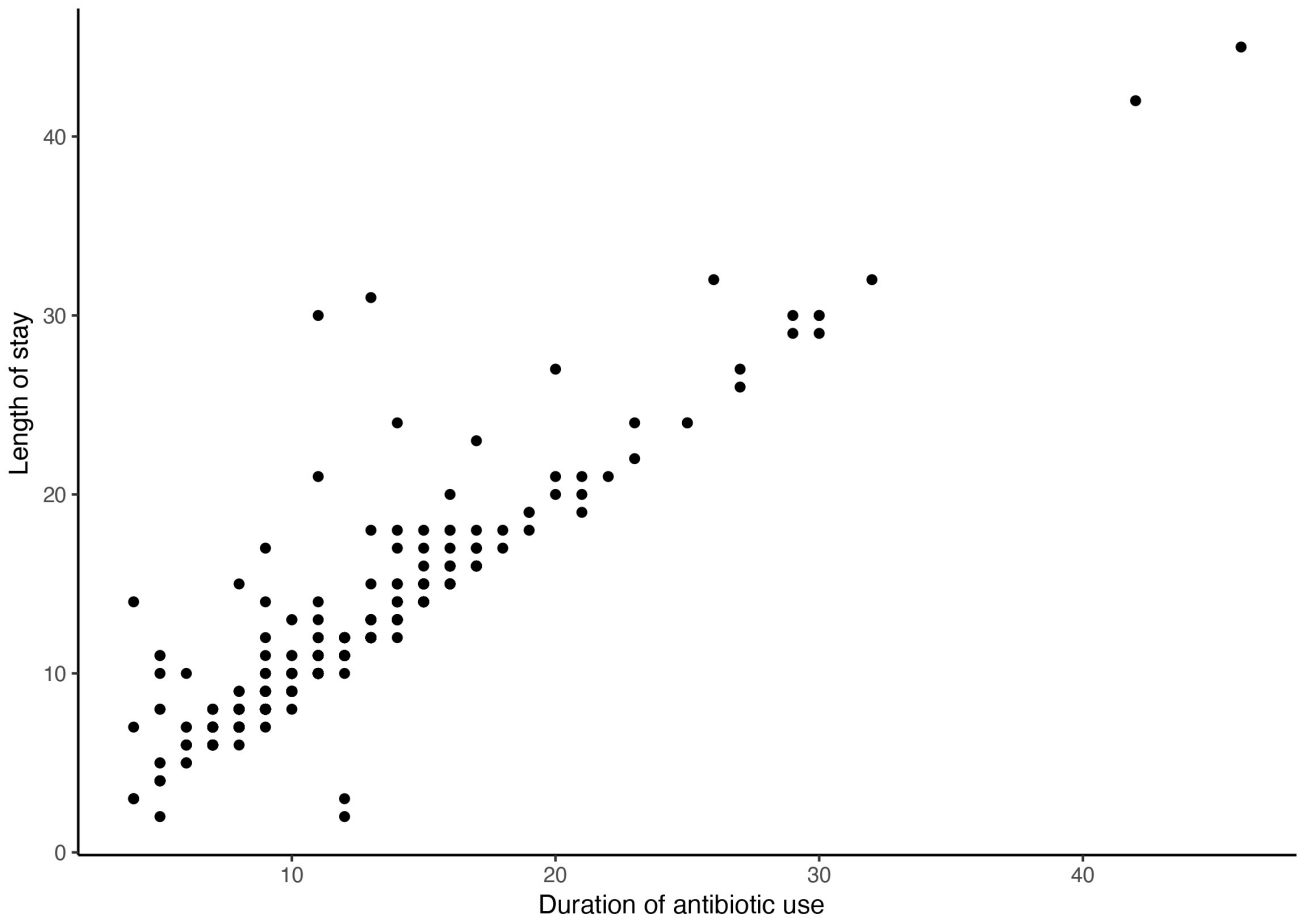
Scatter plot of antibiotic duration vs. length of stay. Scatter distribution of 423 patients illustrating the observed relationship between antibiotic duration and hospital stay.

**Figure 3:**
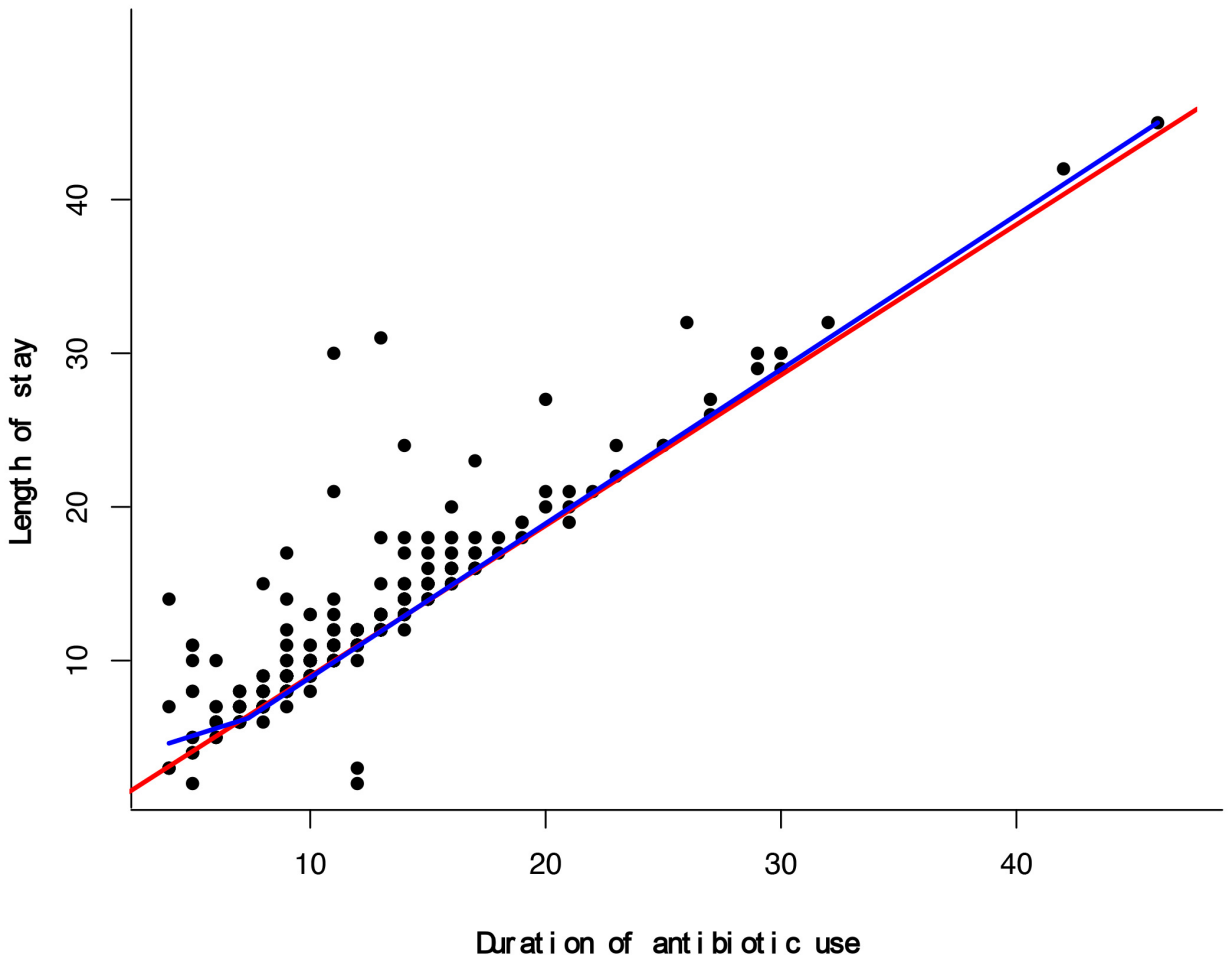
Piecewise regression model. Segmented regression curve (blue) showing improved fit compared with the simple linear model (red). The estimated inflection point is 7.398 days. Shaded areas represent 95% confidence bands for the fitted values, estimated based on the standard errors of the predicted means from the respective regression models.

To further characterize this non-linear pattern, piecewise regression analysis was performed. As shown in the scatter distribution (Figure 2) and supplementary linear fit (Supplementary Figure S1), the simple linear model did not adequately capture the observed relationship. In contrast, the segmented model provided a markedly improved fit, with the regression curve closely aligning with the empirical data (Figure 3).

The estimated inflection point—representing the transition in the slope of LOS growth relative to antibiotic duration—occurred at 7.398 days. The final piecewise model achieved an R^2^ of 0.864 and a residual standard error (RSE) of 1.987, confirming its superior performance compared with the single-slope linear model.

## 4 Discussion

In this retrospective study of hospitalized patients with community-acquired pneumonia (CAP), we identified several clinical factors independently associated with the length of hospital stay (LOS). Among these, the duration of antibiotic therapy demonstrated a clear non-linear association with LOS, with an inflection point at approximately 7.4 days. This observation is consistent with a growing body of evidence supporting shorter and individualized antibiotic courses for CAP (4, 5, 8). Previous studies have demonstrated that extending antibiotic therapy beyond the point of clinical stability does not improve key outcomes such as mortality, readmission, or long-term complications, and may instead be associated with unnecessarily prolonged treatment and extended hospital stay (9, 10). Importantly, the association between prolonged antibiotic duration and extended LOS should be interpreted cautiously due to the potential for reverse causality. In clinical practice, longer therapy is often a consequence of prolonged hospitalization. Patients experiencing delayed clinical stability or secondary complications inherently require extended hospital stays, which in turn dictates longer antibiotic courses. Thus, prolonged antibiotic use in our model likely serves as a marker of complex disease trajectories rather than a causal driver of increased LOS. Besides, the complexity of these trajectories was likely further amplified by the COVID-19 pandemic, which overlapped with our study period. As recent evidence shows that COVID-19 can profoundly alter pulmonary immune responses and trigger severe inflammatory complications (11), this underlying influence serves as a critical contextual factor that may have further delayed clinical stability.

Other variables identified in our model—including respiratory failure, pressure ulcers, elevated blood urea nitrogen (BUN), and changes in antibiotic regimen—were also associated with LOS. These findings are supported by existing literature. Respiratory failure markedly prolongs hospitalization due to increased disease severity and the need for intensive monitoring and supportive treatment (12–14). To optimize such intensive monitoring, emerging non-invasive imaging tools have shown great clinical value in continuously evaluating regional ventilation and real-time respiratory system conditions. Integrating these dynamic respiratory assessments into future predictive models could further refine LOS forecasting for severe CAP cases (15, 16). Patients with pressure ulcers often require prolonged wound management and infection prevention, which further extends recovery time (14). Adjustment of antibiotic therapy during hospitalization was also linked to prolonged LOS, likely indicating initial treatment insufficiency, development of drug resistance, or drug-related adverse effects, all of which delay clinical stability (12, 17).

Elevated BUN has long been recognized as a marker of disease severity in CAP, reflecting impaired renal perfusion, systemic inflammation, or metabolic stress secondary to serious infection. Prior evidence has shown that higher BUN or BUN-based indices are strongly associated with worse clinical outcomes in pneumonia. A recent meta-analysis demonstrated that the BUN/albumin ratio reliably predicts poor prognosis—including ICU admission and mortality—in patients with pneumonia, underscoring the clinical importance of elevated BUN as an indicator of severe disease (18). Similarly, the widely validated CURB-65 score incorporates elevated urea as a core component for identifying high-risk CAP cases, further confirming its prognostic value (19). These findings provide a biologically and clinically plausible explanation for why BUN emerged as an independent predictor of LOS in our model.

From a clinical translation perspective, it is critical to distinguish between inherent markers of disease severity and potentially modifiable predictors in our model. Variables such as respiratory failure and elevated BUN primarily serve as non-modifiable indicators of baseline physiological decompensation; they are essential for early risk stratification but cannot be directly reversed to shorten LOS. Conversely, the development of pressure ulcers and mid-course changes in antibiotic regimens represent modifiable aspects of inpatient management. Categorizing these factors highlights actionable targets: while baseline severity markers help identify high-risk patients at admission, optimizing nursing care to prevent pressure ulcers and ensuring initial antibiotic appropriateness to avoid subsequent regimen changes provide concrete opportunities to reduce hospitalization duration.

The association between traditional Chinese medicine (TCM) treatment and LOS in our study is notable. Previous research suggests that integrated TCM and Western medicine approaches may improve symptoms such as cough and fever and shorten disease duration in CAP patients (20). However, the directionality of this association in our dataset requires cautious interpretation, as the use of TCM may reflect physician preference, local practice patterns, or specific patient characteristics.

This study has several strengths, including the use of real-world clinical data, incorporation of both baseline and in-hospital variables, and the application of non-linear modeling to better characterize complex clinical relationships that may be overlooked by traditional linear analyses. The use of 10-fold cross-validation further enhances the robustness of our findings.

Nevertheless, several limitations must be acknowledged. First, as a single-center retrospective study, the external validity of the predictive model remains unverified; applying these findings to other clinical settings requires prior external validation in independent cohorts. Second, while our 10-fold cross-validation supports internal stability, formal model calibration metrics (e.g., calibration curves) were not systematically evaluated. Third, due to our retrospective design, specific pathogen data, the time gap to targeted antibiotics, and graded complication severity scores were unavailable, which may introduce residual confounding. Fourth, while baseline severity was adjusted for using granular parameters, validated composite scores like CURB-65 and specific ICU labels were unavailable in our dataset. Furthermore, the exclusion of in-hospital deaths inherently introduces survivorship bias. Consequently, our model is exclusively applicable for predicting the LOS of patients who survive to discharge, and it may not fully capture the total healthcare burden or bed utilization associated with CAP non-survivors. Methodologically, while our model captures a high degree of variance (R^2^ = 0.864) and utilizes restricted cubic splines to approximate a random distribution of residuals, the inherent right-skewness of LOS data suggests generalized linear models may be preferable in future studies. Additionally, performing stepwise variable selection prior to the cross-validation loop introduces potential information leakage. Future predictive pipelines should embed penalized regression techniques (such as LASSO) entirely within the validation loop to minimize inferential bias. Because the 7.398-day inflection point was derived as a point estimate, future multicenter studies are needed not only to validate our identified predictors but also to utilize bootstrap resampling to establish formal confidence intervals for this antibiotic duration threshold.

In conclusion, our study identified several key predictors associated with prolonged hospital stay in CAP patients, including respiratory failure, pressure ulcers, elevated BUN, modification of antibiotic therapy, and the duration of antibiotic use. Notably, antibiotic duration exhibited a significant non-linear relationship with LOS, with a turning point at approximately 7.4 days. Rather than implying a direct causal effect, prolonged antibiotic therapy beyond 1 week likely serves as a robust clinical marker of delayed recovery and complex disease trajectories. These findings emphasize the need for careful reassessment of treatment response when antibiotic therapy extends beyond 1 week and highlight the importance of early identification of patients at risk for prolonged hospitalization. Early optimization of treatment strategies may help reduce medical burden and improve outcomes for patients with community-acquired pneumonia.

## Data Availability

The raw data supporting the conclusions of this article will be made available by the authors, without undue reservation.

## Supplementary material

Supplementary Figure 1 Linear regression fit. Simple linear regression model demonstrating the inadequate linear fit between antibiotic duration and hospital stay.
