# Supplementary figures and images for: Predicting length of hospital stay in community-acquired pneumonia using clinical and treatment factors: a retrospective study with restricted cubic spline and piecewise regression analysis
Source: Front Public Health. 2026 Apr 22;14:1768432. doi: 10.3389/fpubh.2026.1768432 (PMC13144054; doi:10.3389/fpubh.2026.1768432)

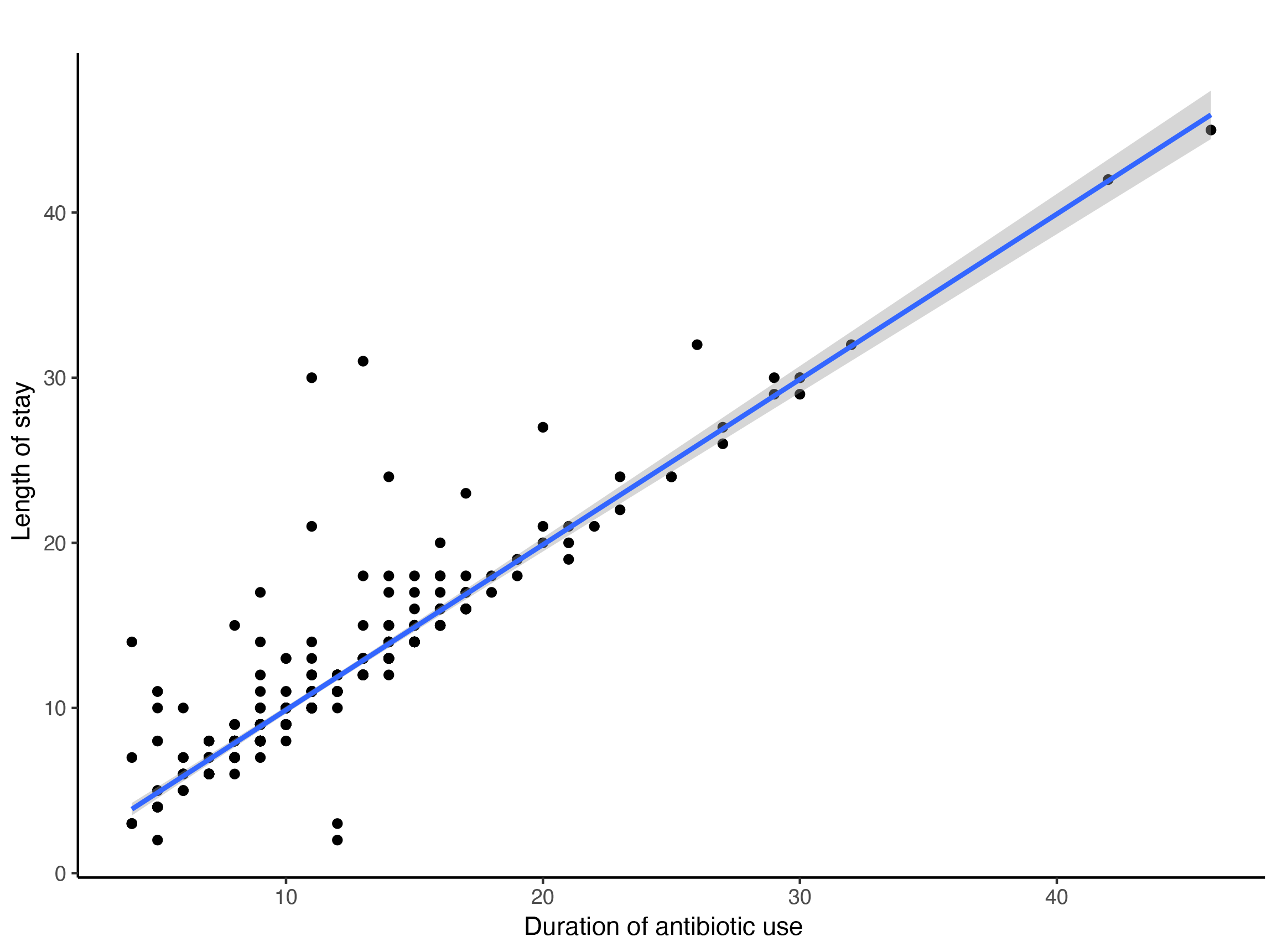

Supplement: Supplementary Figure 1 — Linear regression fit. Simple linear regression model demonstrating the inadequate linear fit between antibiotic duration and hospital stay. [file Image_1.tif]
